# Lymphopenia in Bacterial Sepsis and SARS-CoV-2 Infection

**DOI:** 10.3390/biomedicines14020438

**Published:** 2026-02-15

**Authors:** Raluca Terteşş, Lucian Cristian Petcu, Bogdan Florentin Niţu, Mihaela Mariana Mavrodin, Elena Cucli, Elena Andreea Topa, Constantin Ionescu, Nicolae Cârciumaru, Simona Claudia Cambrea

**Affiliations:** 1 Clinical Hospital of Infectious Diseases Constanţa, Ferdinand Blvd. No. 100, 900709 Constanta, Romaniacucli_elena@yahoo.com (E.C.); topaelena93@gmail.com (E.A.T.); cambrea.claudia@gmail.com (S.C.C.); 2 Faculty of Medicine, Ovidius University Constanţa, Aleea Universității No. 1, Building B, 900470 Constanta, Romania

**Keywords:** sepsis, lymphopenia, neutrophil-to-lymphocyte ratio, prognostic, outcome

## Abstract

**Background:** Sepsis is a life-threatening organ dysfunction that results from an exaggerated host immune response to disseminated infection. The relationship between lymphopenia and sepsis has been extensively studied, and in particular, sepsis-induced lymphopenia is gradually being recognized as an essential factor in the prognosis of sepsis. Notably, sepsis-induced lymphopenia has been associated with worse outcomes, including increased risk of secondary infections, multiple organ failure, and death. Few studies have directly compared the dynamic evolution of lymphocyte counts between different etiologies of sepsis or evaluated their prognostic value using serial measurements. This study aims to explore the temporal dynamics of lymphopenia, but also of neutrophil-to-lymphocyte (NLR) ratio in patients with severe systemic infections and to assess their relationship with in-hospital mortality. **Methods**: A prospective cohort of 95 adult patients was analyzed. Absolute lymphocyte counts (ALCs) and neutrophil-to-lymphocyte (NLR) ratio values were recorded on Days 1, 3, 5, and 7. Comparisons were made between different infectious etiologies and outcomes, and ROC analysis assessed predictive performance. **Results and Conclusions**: Patients with viral sepsis (“COVID-19”) showed a significant and sustained decrease in lymphocyte counts (*p* < 0.001) and a progressive increase in NLR (*p* < 0.001), unlike patients with bacterial sepsis. In correlation with outcome, regardless of etiology, lymphocyte counts were significantly lower in non-survivors from Day 3 onward, while NLR was significantly higher on Day 7 (*p* = 0.002). Early NLR and ALC had limited predictive value, but longitudinal trends were associated with poor prognosis.

## 1. Introduction

Sepsis is a life-threatening organ dysfunction that results from an exaggerated host immune response to disseminated infection [1]. Recent estimates suggest that sepsis affects nearly 50 million people worldwide each year, leading to high mortality rates and huge healthcare costs [2].

It is known that sepsis pathophysiology is characterized (in part) by increased production of both pro- and anti-inflammatory cytokines, with major systemic disturbance, particularly resulting in transient severe lymphopenia and long-lasting immune dysfunction [3]. Lymphopenia, alternatively termed lymphocytopenia, delineates a pathological state characterized by a diminished concentration of lymphocytes in the peripheral blood [4]. The etiology of lymphopenia encompasses a multitude of factors, including infections, but also autoimmune disorders, pharmacological interventions, and exposure to radiation therapy [4].

The relationship between lymphopenia and sepsis has been extensively studied, and in particular, sepsis-induced lymphopenia is gradually being recognized as an essential factor in the prognosis of sepsis [5]. Notably, sepsis-induced lymphopenia has been as-sociated with worse outcomes, including increased risk of secondary infections, multiple organ failure, and death [6,7]. In a comprehensive review, Hotchkiss et al. highlighted that sepsis-induced immunosuppression is associated with increased susceptibility to secondary infections and mortality, supporting the concept that immune paralysis represents a key contributor to adverse outcomes in critically ill patients [8].

Sepsis-induced lymphopenia is usually defined as an absolute lymphocyte count (ALC) of less than 1000 cells/µL [9,10].

The emergence of the “COVID-19” pandemic has further complicated the scenario, resulting in an increased incidence of sepsis cases. This rise is coupled with the pandemic’s tendency to cause lymphopenia, which merits additional investigation to unravel the intricate pathophysiological pathways in sepsis [10].

In viral sepsis, especially with “COVID-19”, lymphopenia is driven by a combination of indirect and direct mechanisms. Indirectly, the cytokine storm and associated hyperlacticaemia impair lymphocyte proliferation and induce apoptosis [11]. The infection also alters hematopoiesis, favoring myeloid over lymphoid differentiation, and promotes a glycolytic shift in metabolism, resulting in elevated ROS (Reactive Oxygen Species) production and further lymphocyte depletion [12].

In bacterial sepsis, similar patterns of lymphopenia emerge, particularly within the CD4^+^ T-cell compartment [3]. Sepsis leads to massive apoptosis of CD4^+^ T-cells and a skewed recovery predominantly through expansion of endogenous memory cells rather than thymic output. Despite numeric recovery, functional impairments persist, including diminished proliferation, cytokine production, and reduced TCR (T-cell receptor) repertoire diversity [3]. CD4^+^ T-cells exhibit global energy and increased expression of inhibitory receptors, weakening their helper function. Furthermore, sepsis distorts CD4^+^ T-cell subset distribution, reducing Th1, Th2, Th17, and Tfh (T follicular helper) cells while increasing regulatory T-cells (Tregs), with uncertain clinical implications [13].

Collectively, these mechanisms highlight a shared immunopathological pathway in sepsis evolution, leading to persistent lymphopenia, impaired T-cell immunity, and in-creased vulnerability to secondary infections and mortality.

Despite these observations, few studies have directly compared the dynamic evolution of lymphocyte counts between different etiologies of sepsis or evaluated their prognostic value using serial measurements. This study aimed to evaluate the temporal dynamics of lymphopenia and neutrophil-to-lymphocyte (NLR) ratio in patients with severe systemic infections and to assess their potential relationship with in-hospital mortality.

## 2. Materials and Methods

### 2.1. Study Design and Population

**Figure 1:**
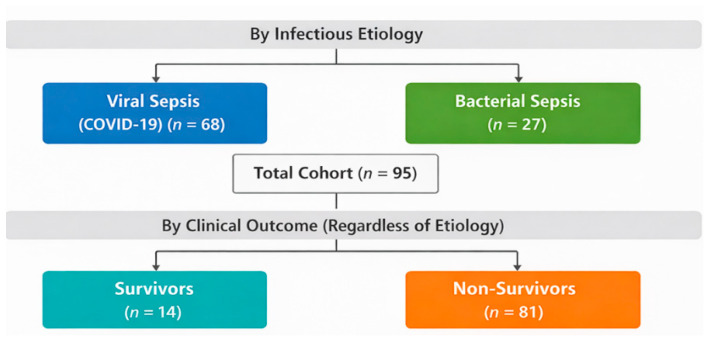
Study cohort stratification.

We conducted a prospective observational study in the Clinical Hospital of Infectious Diseases of Constanta and included adult patients admitted in the Intensive Care Unit of our hospital, during 2021–2025. The study included 95 critically ill patients, diagnosed with sepsis and septic shock; no healthy control group was included and the study focused on the comparison between viral and bacterial etiology of sepsis. The study employed a dual-level stratification approach. First, the study cohort was stratified in two main groups based on confirmed infectious etiology (Figure 1). Subsequently, the total cohort study was further divided based on clinical outcome (Figure 1), regardless of the underlying etiology. This stratification allowed us to explore the prognostic significance of the selected biomarkers. The main criteria for inclusion were fulfilling Sepsis-3 criteria, Definition of Sepsis and Septic Shock, Third Edition (Sepsis-3) published in 2016 [1]. The criteria for sepsis diagnosis were (1) identification of suspected infection; (2) presence of a sequential organ failure score (SOFA) change ≥ 2 points. Patients were followed until discharge or in-hospital death.

Most patients were infected during the Delta period, as confirmed by epidemiological surveillance data. The cohort imbalance reflects clinical realities during the study period, when “COVID-19” ICU admissions greatly outnumbered other etiologies of sepsis cases.

Regarding the etiology in the bacterial sepsis cohort, the most frequently isolated pathogens were *Klebsiella pneumoniae* and *Staphylococcus aureus*, as we can see in Table 1.

**Table 1:** Frequency of pathogens.

Pathogen	Frequency
*Klebsiella pneumoniae*	37.0%
*Staphylococcus aureus*	37.0%
*Acinetobacter baumannii*	29.6%
*Streptococcus pneumoniae*	11.1%
*Escherichia coli*	11.1%
*Pseudomonas aeruginosa*	7.4%
*Stenotrophomonas maltophila*	7.41%
*Enterobacter* spp.	3.7%
*Serratia* spp.	3.7%
*Clostridium difficile*	2.94%

Less frequently encountered organisms included *Pseudomonas aeruginosa*, *Stenotrophomonas maltophilia*, *Enterobacter* spp., *Serratia* spp., and *Clostridium difficile*. The most common sources were respiratory ones, as we can see in Table 2.

**Table 2:** Source of pathogens.

Source	Frequency
Respiratory secretions	36.8%
Blood cultures	8.4%
Urine samples	5.3%
Cerebrospinal fluid (CSF)	2.1%
Stool samples (for Clostridium)	2.1%

These data suggest that the pulmonary site was the dominant source of sepsis in the bacterial cohort, followed by bloodstream and urinary tract infections.

Inclusion Criteria:

1. Age—patients aged ≥ 18 years.
2. Sepsis Diagnosis—sepsis defined according to Sepsis 3: suspected or confirmed infection plus an acute increase in SOFA score by ≥2 points [1].
3. Positive Etiology:
  ○ Viral sepsis (COVID-19): positive RT-PCR for SARS-CoV-2.
  ○ Bacterial sepsis: positive culture from blood, sputum, or urine yielding a typical bacterial pathogen.
4. Informed Consent—written informed consent signed by the patient or their legal representative.

Exclusion Criteria:

1. Chronic Immunosuppression:
  ○ active chemotherapy or radiotherapy within the past 6 months.
  ○ active hematologic malignancy.
  ○ HIV infection.
2. Transplantation, solid organ or hematopoietic stem cell transplant recipients:
  ○ treatment with monoclonal antibodies or other potent immunomodulators.
3. Hospital stay shorter than 24 h, insufficient for longitudinal sampling.
4. Special Clinical Conditions:
  ○ pregnancy.
5. Refusal of Procedures:
  ○ patient or representative declines repeated blood sampling.

The study was approved by the Ethics Committee of Infectious Diseases Hospital of Constanta, in accordance with the Declaration of Helsinki No. 12/01.09.2021, and in-formed consent was obtained from all participants or their legal representatives.

### 2.2. Data Collection and Variables

Clinical and demographic data were collected, including age, sex, length of hospitalization, and in-hospital survival status.

Laboratory parameters were assessed on Days 1, 3, 5, and 7 of hospitalization:

- Absolute lymphocyte count (ALC).
- Neutrophil-to-lymphocyte ratio (NLR).

And also peripheral T-cell subsets (CD3^+^, CD4^+^, CD8^+^ counts, CD4^+^/CD8^+^ ratio) were measured at admission for 35 patients.

Complete blood count (CBC), including total leukocyte, neutrophil, and lymphocyte counts, as well as the automatically calculated neutrophil-to-lymphocyte ratio (NLR), were performed using a Mindray B C 6200 hematology analyzer (Mindray Bio-Medical Electronics Co., Ltd., Shenzhen, China).

Quantification of T-cell subsets (CD3^+^, CD4^+^, CD8^+^) and calculation of the CD4^+^/CD8^+^ ratio were performed by flow cytometry using the Beckman Coulter AQUIOS Flow Cytometer (Beckman Coulter, Miami, FL, USA).

### 2.3. Statistical Analysis

In our study, the selected biomarkers were assessed at four time points, and none met the assumption of normality. Between group comparisons at each time point were performed using the Mann–Whitney U test. Mortality during follow up resulted in partially overlapping samples without complete repeated measures data, making paired non-parametric tests (e.g., Friedman) and mixed effects models inappropriate.

Therefore, the four time points were treated as independent samples within each group, and temporal differences were assessed using the Kruskal–Wallis H test. Post hoc tests were performed only when the global test was significant, with Bonferroni adjustment. Missing data, occurring exclusively due to death, were not imputed and were handled using an available case approach.

ROC analysis was used to evaluate the prognostic performance of the selected biomarkers at admission. Kaplan–Meier survival curves were generated, and hazard ratios (HRs) with 95% confidence intervals (CIs) were calculated to assess the relative mortality risk between groups. A two-tailed *p*-value < 0.05 was considered statistically significant.

## 3. Results

### 3.1. Cohort Characteristics

Patients with “COVID-19” (n = 68) had a mean age of 66.5 ± 11.5 years (median 68.5; IQR 61.0–74.0), compared to 65.1 ± 13.1 years (median 67.0; IQR 57.0–75.0) in the bacterial sepsis group (n = 27). Levene’s test confirmed equal variances (F = 0.276, *p* = 0.601), and an independent samples t-test showed no significant difference in mean age between groups (t = 0.512, df = 93, *p* = 0.610; mean difference = 1.40 years; 95% CI: –4.02 to 6.81).

Regarding gender distribution, in the viral sepsis group, 54.4% (37/68) were male and 45.6% (31/68) were female, whereas in the bacterial sepsis group, 77.8% (21/27) were male and 22.2% (6/27) were female. A chi-square test of association confirmed a relationship between cohort and sex (χ^2^ = 4.52, df = 1, *p* = 0.035), indicating a higher proportion of males in the bacterial sepsis cohort.

**Figure 2:**
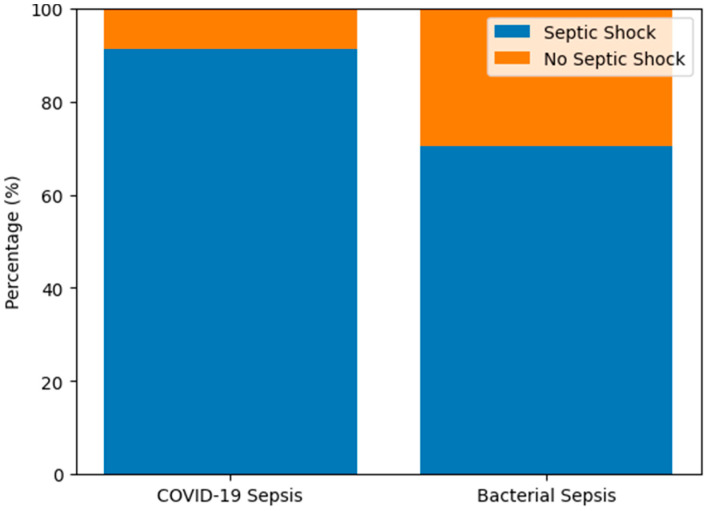
Distribution of septic shock by etiology.

Regarding the severity of sepsis cases, among patients with viral sepsis (“COVID-19”), 91.18% developed septic shock, compared to 70.37% in the bacterial sepsis group (Figure 2). The odds of developing septic shock were 4.35 times higher in the “COVID-19” cohort compared with patients with bacterial sepsis (OR = 4.351, 95% CI = 1.341–14.112). Similarly, the relative risk (RR) of septic shock in the “COVID-19” group was 1.296 (95% CI = 1.003–1.673), indicating a higher probability of severe hemodynamic compromise in viral sepsis. This finding is consistent with a higher burden of circulatory failure in viral sepsis.

A cross-tabulation analysis was performed to assess the association between sepsis etiology (“COVID-19” vs. bacterial) and the occurrence of septic shock. The Chi-square test demonstrated a statistically significant relationship between the two variables (χ^2^ = 6.658, df = 1, *p* = 0.010), indicating that the distribution of septic shock differed significantly between groups.

Consistent with the higher prevalence of septic shock, patients with COVID-19-associated sepsis more frequently required advanced organ support, including vasopressor therapy and mechanical ventilation, compared with those with bacterial sepsis (Table 3).

**Figure 3:**
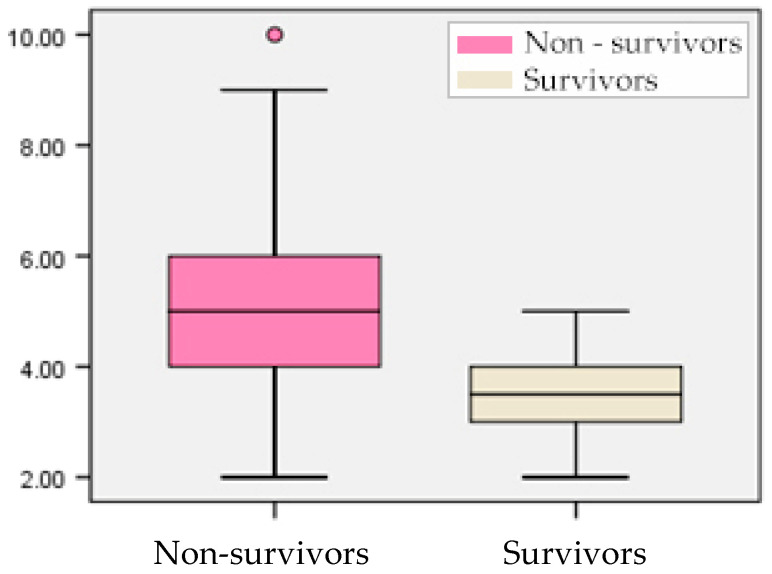
Box-plot representation of SOFA score according to outcome.

Regarding the clinical severity scores at ICU admission according to outcome, the median SOFA score was significantly higher among non-survivors compared to survivors (5.00 versus 3.50, *p* < 0.001), indicating greater organ dysfunction severity in patients who did not survive (Figure 3). Over half of the non-survivors (53.1%) presented with SOFA scores between 4 and 5, while none of the survivors had values above 5, underscoring the prognostic value of the SOFA score in early mortality risk assessment.

**Figure 4:**
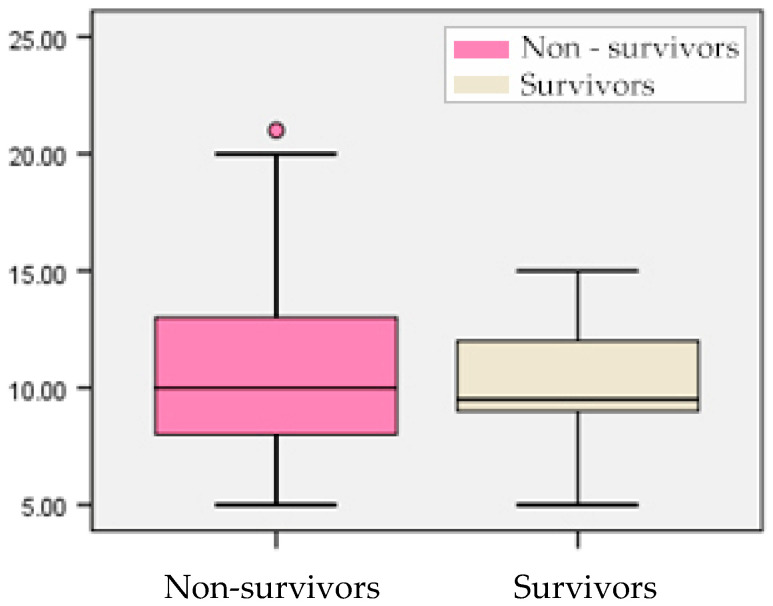
Box-plot representation of APACHE II score according to outcome.

In contrast, the APACHE II score did not differ significantly between survivors and non-survivors (median 9.50 vs. 10.00, *p* = 0.642) (Figure 4). Both groups exhibited overlapping score ranges (5–21 vs. 5–15), suggesting limited discriminative power of APACHE II in this cohort.

### 3.2. Outcome Measures

**Figure 5:**
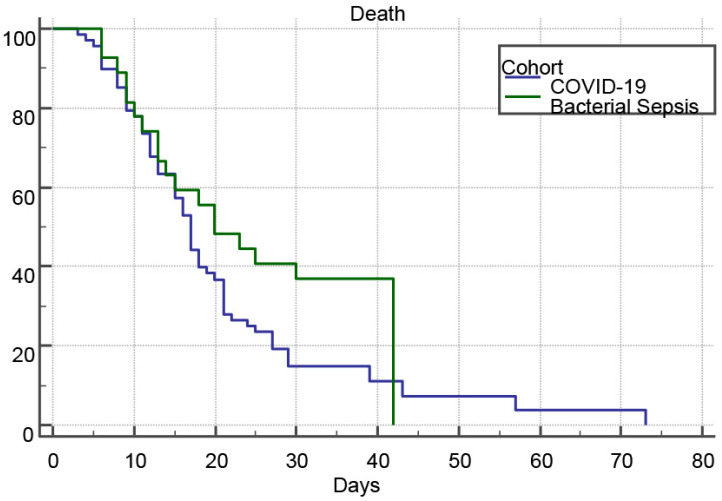
Kaplan–Meier survival curves for the COVID-19 and Bacterial Sepsis groups.

The primary outcome was in-hospital mortality. Survival analysis using Kaplan–Meier curves revealed that 91.2% (62/68) of viral sepsis (“COVID-19”) patients and 70.4% (19/27) of bacterial sepsis patients experienced in-hospital mortality (Figure 5). Median survival times were 17.0 days (95% CI, 15.0–19.0) for the viral sepsis cohort and 20.0 days (95% CI, 13.0–42.0) for the bacterial sepsis cohort, with mean survival times of 19.21 days (95% CI, 16.57–21.85) and 24.82 days (95% CI, 19.45–30.18), respectively. The log rank test indicated no significant difference in survival distributions between groups (χ^2^ = 2.7003, df = 1, *p* = 0.1003), and Cox proportional hazards modeling yielded a hazard ratio of 1.50 (95% CI, 0.94–2.40), further demonstrating comparable in hospital death risk in severe “COVID-19” versus bacterial sepsis.

### 3.3. Lymphocyte Count and T-Cell Subsets

The Mann–Whitney U test was applied to compare lymphocyte counts between patients with viral (“COVID-19”) and bacterial sepsis at different time points (D1, D3, D5, D7) (Table 4). No statistically significant differences were found between the two groups at any time point. On Day 1 (D1), median lymphocyte levels were slightly higher in the “COVID-19” group (Median = 660 cells/µL) compared to the bacterial sepsis group (Median = 580 cells/µL), but the difference did not reach statistical significance (U = 796.000, Z = −1.007, *p* = 0.314) (Table 4). By Day 3 (D3), median values decreased in the “COVID-19” group (525 cells/µL) and remained comparable to bacterial sepsis (650 cells/µL; U = 744.500, Z = −1.432, *p* = 0.152) (Table 4). On Day 5 (D5), lymphocyte counts continued to decline in both groups, with persistently lower levels in the “COVID-19” cohort (Median = 480 versus 550 cells/µL; U = 752.500, Z = −1.173, *p* = 0.241) (Table 4). By Day 7 (D7), the difference remained non-significant (U = 567.000, Z = −1.860, *p* = 0.063) (Table 4), although the bacterial sepsis group showed a trend toward higher lymphocyte recovery compared to “COVID-19”. Overall, lymphocyte counts were consistently lower in patients with viral sepsis (“COVID-19”) throughout the observed period, but the differences compared to bacterial sepsis did not reach statistical significance. The observed trend may reflect the prolonged lymphopenia associated with viral-induced immune dysregulation.

The Kruskal–Wallis analysis demonstrated a statistically significant change in lymphocyte counts across the assessed time points within the “COVID-19” group (H = 52.241, df = 4, *p* = 0.001), indicating a non-random temporal variation in lymphocyte levels during hospitalization (Table 5). In contrast, no significant temporal change was observed within the bacterial sepsis group (H = 3.205, df = 4, *p* = 0.524), suggesting relatively stable lymphocyte counts over time in this cohort (Table 5).

The analysis of peripheral blood T-cell subsets (CD3^+^, CD4^+^, CD8^+^) and the CD4^+^/CD8^+^ ratio on admission was determined in the case of 35 patients.

The Mann–Whitney U test demonstrated no statistically significant differences between the two groups regarding CD3^+^, CD4^+^, or CD8^+^ T-cell counts (Table 6). Median CD4^+^ counts were slightly higher in the “COVID-19” cohort (Median = 128 cells/mm^3^) compared to bacterial sepsis (Median = 100 cells/mm^3^; U = 76.000, Z = −0.908, *p* = 0.364) (Table 6). Similarly, CD8^+^ levels showed no significant variation between “COVID-19” (Median = 88 cells/mm^3^) and bacterial sepsis (Median = 121 cells/mm^3^; U = 72.500, Z = −1.052, *p* = 0.293) (Table 6). Total CD3^+^ counts also did not differ significantly between groups (Median = 240 cells/mm^3^ in “COVID-19” vs. 370 cells/mm^3^ in bacterial sepsis; U = 66.500, Z = −1.299, *p* = 0.194) (Table 6). However, a statistically significant difference was observed for the CD4^+^/CD8^+^ ratio, which was significantly higher in the “COVID-19” group (Median = 1.67) compared to bacterial sepsis (Median = 0.60; U = 32.000, Z = −2.722, *p* = 0.006) (Table 6). These findings suggest that while absolute T-cell counts (CD3^+^, CD4^+^, CD8^+^) are similarly reduced in both etiologies, the CD4^+^/CD8^+^ imbalance is more pronounced in viral sepsis, reflecting differential patterns of T-cell dysregulation and immune exhaustion characteristic of “COVID-19”-associated lymphopenia.

### 3.4. Neutrophil-to-Lymphocyte Ratio (NLR)

On Day 1 (D1), the NLR median values were comparable between the two groups (“COVID-19”: 10.5 versus bacterial sepsis: 12.02; U = 821.000, Z = −0.800, *p* = 0.423), indicating no significant difference at baseline (Table 7). By Day 3 (D3), the NLR increased in both groups, with slightly higher median values in “COVID-19” (16.0) compared to bacterial sepsis (13.10), but the difference remained statistically non-significant (U = 788.500, Z = −1.069, *p* = 0.285) (Table 7). On Day 5 (D5), the upward trend persisted (“COVID-19” median = 24.79 versus bacterial sepsis = 19.87), though still without statistical significance (U = 718.000, Z = −1.464, *p* = 0.143) (Table 7). By Day 7 (D7), however, the Mann–Whitney U test showed a statistically significant difference between groups (U = 501.000, Z = −2.487, *p* = 0.013), with higher NLR values in “COVID-19” (Median = 29.28) compared to bacterial sepsis (Median = 15.36) (Table 7). These results indicate that while both groups exhibited an increasing NLR over time, the progression was significantly more pronounced in viral sepsis (“COVID-19”). The delayed but sustained rise in NLR in “COVID-19” patients could suggest a prolonged inflammatory and immunoregulatory imbalance, possibly reflecting persistent cytokine activation and impaired lymphocyte recovery.

The Kruskal–Wallis test (Table 8) revealed a statistically significant change in neutrophil-to-lymphocyte ratio (NLR) across the evaluated time points within the “COVID-19” group (H = 81.801, df = 3, *p* = 0.001), indicating a non-random temporal evolution of NLR during hospitalization. In contrast, no statistically significant temporal variation in NLR was observed within the bacterial sepsis group (H = 6.887, df = 3, *p* = 0.076), suggesting relatively stable NLR values over time in this cohort (Table 8).

### 3.5. Prognostic Value of Lymphocyte and NLR in Correlation with Outcome

The second stratification of the study cohort was based on the outcome, in order to explore the prognostic significance of selected biomarkers.

#### 3.5.1. Lymphocyte Count and Outcome

The Mann–Whitney U test revealed no statistically significant difference in lymphocyte counts between survivors and non-survivors on Day 1 (Median = 660 vs. 585 cells/μL, *p* = 0.505) (Table 9). From Day 3 onward, however, lymphocyte counts were significantly lower in non-survivors. On Day 3, the median lymphocyte count was 520 cells/μL in non-survivors and 710 cells/μL in survivors (*p* = 0.013) (Table 9). The difference became more pronounced on Day 5 (460 vs. 685 cells/μL, *p* = 0.001) and Day 7 (485 vs. 805 cells/μL, *p* < 0.001) (Table 9). Serial comparisons over time indicated that lymphocyte counts decreased progressively and significantly in non-survivors (*p* < 0.001), whereas in survivors, lymphocyte values remained relatively stable throughout the observation period (*p* = 0.242) (Table 10). These findings indicate that persistent and worsening lymphopenia is associated with unfavorable outcomes in sepsis across etiologies. The early divergence in lymphocyte trajectories observed from Day 3 onward suggests that longitudinal assessment of lymphocyte counts may provide additional prognostic information beyond single time-point measurements in critically ill septic patients.

#### 3.5.2. NLR and Outcome

The Mann–Whitney U test showed that NLR values did not differ significantly between survivors and non-survivors during the initial five days of hospitalization (*p* > 0.05) (Table 11). On Day 1, median NLR values were comparable (10.51 vs. 11.49, *p* = 0.324), and similar non-significant differences were observed on Days 3 (16.20 vs. 13.25, *p* = 0.159) and 5 (25.56 vs. 14.46, *p* = 0.093) (Table 11). However, by Day 7, a statistically significant difference emerged, with non-survivors exhibiting substantially higher NLR values compared to survivors (Median = 29.76 vs. 11.01, *p* = 0.002) (Table 11). Across successive time points, NLR values increased progressively and significantly among non-survivors (*p* < 0.001), whereas no significant temporal change was observed in survivors (*p* = 0.612) (Table 12). This delayed but sustained elevation of NLR among non-survivors is consistent with persistent inflammatory imbalance and immune dysregulation, potentially reflecting ongoing neutrophil predominance and impaired lymphocyte recovery. Collectively, these findings suggest that NLR behaves as a dynamic biomarker, with prognostic information becoming more apparent over the course of illness, thereby supporting the concept that serial assessment may be more informative than single early measurements in septic patients.

#### 3.5.3. Predictive Performance of ALC, NLR and T-Cell Subsets

**Figure 6:**
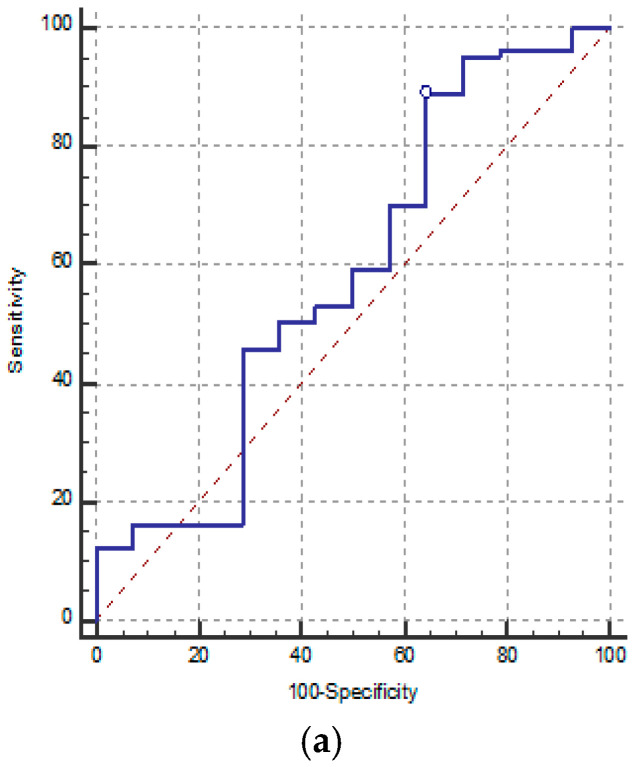

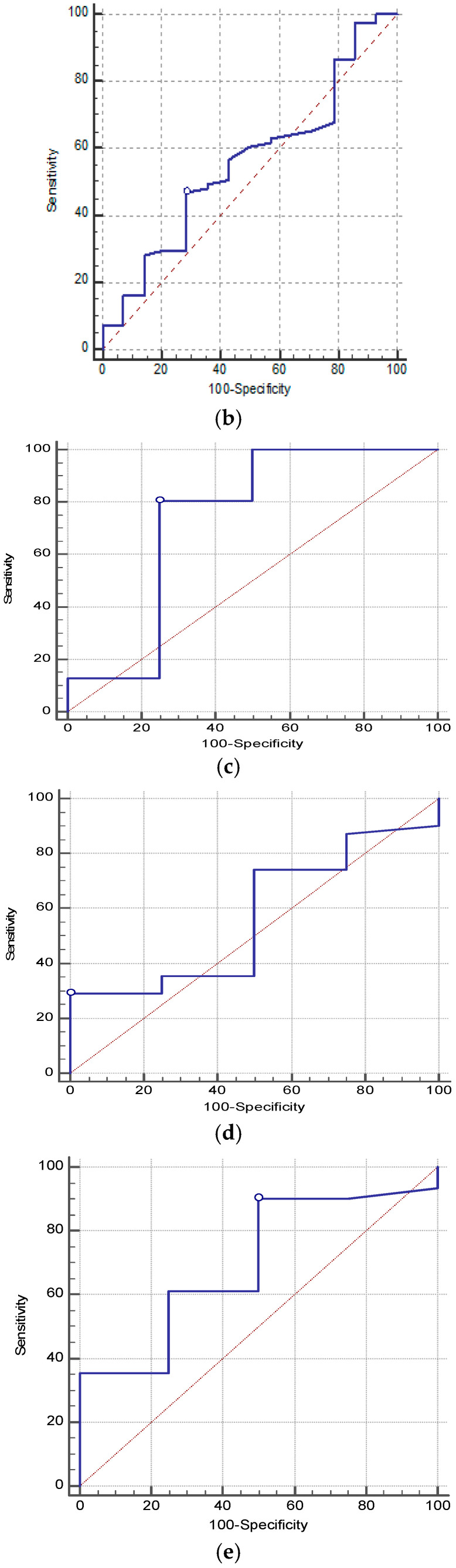
(**a**) ROC curve for NLR. (**b**) ROC curve for ALC. (**c**) ROC curve for CD4^+^. (**d**) ROC curve for CD8^+^. (**e**) ROC curve for CD4^+^/CD8^+^.

Receiver operating characteristic (ROC) analysis was performed to evaluate the prognostic performance of neutrophil-to-lymphocyte ratio (NLR), absolute lymphocyte count (ALC), and T-cell subsets (CD4^+^, CD8^+^, and CD4^+^/CD8^+^ ratio) at admission for predicting in-hospital mortality (Table 13, Figure 6a–e). Overall, none of the evaluated biomarkers demonstrated statistically significant discriminative ability. The AUC values ranged from 0.556 to 0.734, and in all cases the 95% confidence intervals included or closely approached the null value of 0.5, with non-significant z-test results (all *p* > 0.05). NLR and ALC exhibited limited prognostic performance, with low AUC values (0.583 and 0.556, respectively), indicating poor discrimination between survivors and non-survivors. Although selected cut-off values yielded relatively high sensitivity, specificity remained low, limiting their clinical applicability as standalone predictors. Among T-cell parameters, CD4^+^ T-cell count showed the highest AUC (0.734), while the CD4^+^/CD8^+^ ratio yielded an AUC of 0.698; however, neither reached statistical significance, reflecting only modest and non-confirmatory discriminative capacity. CD8^+^ T-cell counts demonstrated particularly weak performance, with an AUC of 0.569, consistent with a prognostic value close to random classification.

Taken together, these findings indicate that baseline measurements of NLR, ALC, and T-cell subsets lack statistically significant prognostic accuracy for mortality prediction when assessed individually at admission, underscoring the limitations of single time-point immunological markers and supporting the need for dynamic or combined prognostic approaches.

## 4. Discussion

Immunoparalysis is increasingly recognized as a key marker in the prognostic assessment of systemic infections. In this prospective observational study directly comparing viral and bacterial systemic infections, we described the longitudinal dynamics of lymphocyte counts and NLR, and analyzed their prognostic significance. Our findings are built on recent literature and explore novel facets of immune dysregulation in sepsis of various etiology. In our analysis, patients with viral sepsis (“COVID-19”) demonstrated a more profound and sustained decline in absolute lymphocyte counts compared to the bacterial etiology cohort. This finding aligns with current evidence indicating that SARS-CoV-2 infection induces extensive lymphocyte apoptosis, bone marrow suppression, and T-cell exhaustion, thereby contributing to immune dysfunction and unfavorable clinical outcomes [10,14].

Although absolute T-cell subset counts (CD3^+^, CD4^+^, CD8^+^) did not differ significantly between cohorts, the elevated CD4^+^/CD8^+^ ratio in “COVID-19” (1.65 vs. 0.84, *p* = 0.006) suggests skewed T-cell homeostasis, in line with reports of T-cell apoptosis and exhaustion in SARS-CoV-2 infection [14,15]. In this context, lymphocyte counts below 489 cells/µL and the T-cell lymphopenia below 593 cells/µL, as well as the CD4^+^ below 326 cells/µL had worse outcomes than patients with higher values [16]. Therefore, T-lymphocyte counts as well as CD4^+^T-cell counts at the beginning of the hospitalization can provide valuable information for targeted therapy to septic patients who are inclined to develop complications [16].

The neutrophil-to-lymphocyte (NLR) ratio has emerged as a dynamic inflammation marker. A recent meta-analysis confirmed that higher value of NLR is associated with poor prognosis in adult sepsis (HR 1.69; 95% CI: 1.43–1.99) [17], and our data showed that NLR rose from a median of 10.5 on Day 1 to 29.3 by Day 7 in “COVID-19”, compared with a modest increase in bacterial sepsis, with a significant intergroup difference at Day 7 (*p* = 0.013). Multiple meta-analyses and cohort studies have confirmed the prognostic utility of NLR in “COVID-19”, with values > 6 consistently associated with ICU admission and in-hospital death [18,19]. In particular, thresholds such as 7.8 and 15.2 have been linked to 5–15-fold increased mortality risk [18,19,20].

Over time, in correlation with survival, NLR increased progressively in non-survivors (*p* < 0.001), while survivors showed no significant change (*p* = 0.612). Our observations are sustaining the results of previous studies that are highlighting NLR as a sensitive biomarker reflecting ongoing systemic inflammation and immunological imbalance in critically ill septic patients [20]. Elevated NLR has been consistently associated with poor clinical outcomes in various infectious and inflammatory conditions, notably in severe “COVID-19”, reflecting immune dysregulation [21,22,23,24,25].

Moreover, time-weighted average NLR (TWA-NLR) has been shown to independently predict 90-day mortality in septic patients [26] supporting the utility of serial rather than single measurements.

In contrast, absolute lymphocyte counts alone did not provide substantial prognostic significance, which might be explained by the variable kinetics of lymphocyte depletion among individuals. This finding is consistent with previous reports showing that lymphocyte counts, although indicative of immunosuppression and poor prognosis when persistently low, might require integration with additional inflammatory markers to achieve adequate predictive accuracy [27,28]. Indeed, the combination of lymphocyte counts with inflammatory markers such as C-reactive protein (CRP), ferritin, or interleukin-6 has been reported to markedly improve predictive models [27,28].

Emerging evidence also suggests that combined markers may outperform single indices: the NLR_NPR (neutrophil-platelet ratio) composite yielded an AUC superior to SOFA score for 28-day mortality prediction in sepsis (AUC 0.56 vs. 0.50) [29]. This highlights the potential of integrated scores incorporating NLR, platelet counts, and possibly other parameters (e.g., IL-6, suPAR) to enhance risk stratification.

Our study is among the few to directly compare lymphopenia across cohorts with “COVID-19” and bacterial sepsis, allowing for the exploration of whether immune alterations are disease-specific or reflect a more generalized immunopathological response. Although the relatively small sample size limits the generalizability of our findings, the observed trends suggest a potential role for CD4^+^/CD8^+^-based indices in outcome assessment, particularly in settings characterized by immune dysregulation.

The dynamic pattern observed for NLR underscores its relevance as a readily available marker when assessed longitudinally rather than at a single time point. This finding highlights the importance of continuous biomarker monitoring and supports the concept that temporal trajectories may be more informative than isolated early measurements.

Importantly, these results should be regarded as hypothesis-generating. While sequential NLR assessment appears to provide additional prognostic information, its use as a standalone predictor is limited, and lymphocyte counts are unlikely to be sufficient unless interpreted in conjunction with other inflammatory and clinical parameters.

From a research perspective, future studies integrating serial hematologic biomarkers with microbiological surveillance and adjusted longitudinal models may help clarify their combined prognostic value. Such approaches may contribute to the development of refined risk stratification frameworks, rather than immediate changes in clinical practice.

## 5. Conclusions

In this study, we evaluated the dynamic evolution and prognostic significance of absolute lymphocyte count and neutrophil-to-lymphocyte ratio (NLR) in patients with severe systemic infections of both viral and bacterial etiology. Our findings indicate that progressive lymphopenia, particularly from Day 3 onward, and a rising NLR trajectory, especially evident by Day 7, were associated with worse outcome.

While both biomarkers registered changes at baseline in all patients, their temporal dynamics differed between groups. Viral sepsis (“COVID-19”) was characterized by a sustained decline in lymphocyte counts and a marked elevation in NLR, reflecting a pattern of immune exhaustion and hyperinflammation not observed in bacterial sepsis. Regarding the outcome, these changes were significantly more pronounced in non-survivors.

Although no statistically significant difference in overall survival was observed between the two etiologies in our cohort, distinct immune trajectories were identified, which may carry prognostic relevance.

Taken together, these findings may suggest that serial monitoring of lymphocyte counts and NLR, rather than reliance on single time-point measurements, may provide additional prognostic information and complement clinical risk stratification, particularly in resource-limited settings. Further studies in larger cohorts using adjusted longitudinal models are warranted to confirm these observations.

### Limitations

This study has several limitations that should be acknowledged. First, due to its prospective observational design, analyses across multiple time points were interpreted as exploratory and descriptive. The modest sample size limited the feasibility of multivariable adjustment and formal longitudinal mixed-effects modeling; therefore, the findings should be regarded as hypothesis-generating rather than confirmatory.

Second, the relatively small cohort size, particularly in the bacterial sepsis group, reduces statistical power and limits the generalizability of the results. The imbalance between groups reflects ICU admission dynamics during the “COVID-19” pandemic rather than deliberate selection bias.

Third, T-cell subset measurements (CD3^+^, CD4^+^, CD8^+^) were available only in a subset of patients, restricting the ability to draw definitive conclusions regarding their prognostic value across the entire cohort.

Future studies in larger, more balanced populations with standardized longitudinal data collection and appropriately adjusted models are warranted to better define the independent prognostic significance of NLR and lymphocyte dynamics in sepsis of different etiologies.

## Figures and Tables

**Table 3:** Organ support requirements by sepsis etiology.

Organ Support	COVID-19-Associated Sepsis (n = 68)	Bacterial Sepsis (n = 27)	Statistical Test	*p*-Value
Vasopressor therapy, n (%)	62 (91.2%)	19 (70.4%)	Chi-square test	0.010
Oxygen therapy, n (%)	68 (100%)	24 (88.9%)	Chi-square test	0.005
Mechanical ventilation, n (%)	62 (91.2%)	19 (70.4%)	Chi-square test	0.010

**Table 4:** Mann–Whitney U Test Comparing Lymphocyte Counts Between COVID-19 and Bacterial Sepsis.

	Study Cohort	N	Mean	Median	SD	Mann–Whitney U	Z	*p*
Lymphocyte D1 (cells/microL)	COVID-19	68	782.63	660.00	447.46	796.000	−1.007	0.314
	BS	27	657.78	580.00	307.44			
Lymphocyte D3 (cells/microL)	COVID-19	68	567.71	525.00	249.74	744.500	−1.432	0.152
	BS	27	685.56	650.00	353.57			
Lymphocyte D5 (cells/microL)	COVID-19	66	491.82	480.00	228.32	752.500	−1.173	0.241
	BS	27	592.41	550.00	344.37			
Lymphocyte D7 (cells/microL)	COVID-19	61	494.26	500.00	200.83	567.000	−1.860	0.063
	BS	25	645.20	580.00	319.62			

**Table 5:** Within-Group Kruskal–Wallis Analysis of Temporal Changes in Lymphocyte Counts in COVID-19 and Bacterial Sepsis.

Lymphocyte (Cells/MicroL)	Kruskal–Wallis H	df	*p*
COVID-19	52.241	4	0.001
BS	3.205	4	0.524

**Table 6:** Mann–Whitney U Test Comparing T-cell subsets Between COVID-19 and Bacterial Sepsis.

	Cohort	N	Mean	Median	SD	Mann–Whitney U	Z	*p* (Two-Tailed)
CD4^+^ (cells/mm^3^)	COVID-19	28	157.11	128.00	99.38	76.000	−0.908	0.364
	BS	7	105.71	100.00	42.45			
CD8^+^ (cells/mm^3^)	COVID-19	28	103.61	88.00	63.45	72.500	−1.052	0.293
	BS	7	144.14	121.00	96.92			
CD3^+^ (cells/mm^3^)	COVID-19	28	296.68	240.00	190.45	66.500	−1.299	0.194
	BS	7	383.71	370.00	176.37			
CD4^+^/CD8^+^ (cells/mm^3^)	COVID-19	28	1.65	1.67	0.75	32.000	−2.722	0.006
	BS	7	0.84	0.60	0.44			

**Table 7:** Mann–Whitney U Test Comparing NLR Between COVID-19 and Bacterial Sepsis.

	Cohort	N	Mean	Median	SD	Mann−Whitney U	Z	*p* (Two-Tailed)
NLR D1	COVID-19	68	10.85	10.50	6.36	821.000	−0.800	0.423
	BS	27	13.15	12.02	9.02			
NLR D3	COVID-19	68	19.56	16.00	13.69	788.500	−1.069	0.285
	BS	27	17.94	13.10	13.98			
NLR D5	COVID-19	66	28.08	24.79	15.94	718.000	−1.464	0.143
	BS	27	23.39	19.87	16.28			
NLR D7	COVID-19	61	30.90	29.28	16.05	501.000	−2.487	0.013
	BS	25	22.45	15.36	19.00			

**Table 8:** Within-Group Kruskal–Wallis Analysis of Temporal Changes in NLR in COVID-19 and Bacterial Sepsis.

NLR	Kruskal–Wallis H	df	*p*
COVID-19	81.801	3	0.001
BS	6.887	3	0.076

**Table 9:** Mann–Whitney U Test Comparing Lymphocyte Counts Between Survivors and Non-Survivors.

Moment	Outcome	N	Mean	Median	SD	Mann–Whitney U	Z	*p* (Two-Tailed)
D1	Non-survivors	81	737.52	660.00	357.42	503.500	−0.667	0.505
	Survivors	14	657.86	585.00	341.88			
D3	Non-survivors	81	551.64	520.00	229.83	330.000	−2.489	0.013
	Survivors	14	724.36	710.00	214.10			
D5	Non-survivors	79	462.91	460.00	198.94	241.000	−3.353	0.001
	Survivors	14	693.21	685.00	224.84			
D7	Non-survivors	72	479.86	485.00	188.76	202.500	−3.528	0.001
	Survivors	14	795.00	805.00	285.76			

**Table 10:** Within-Group Kruskal–Wallis Analysis of Temporal Changes in Lymphocyte Counts in Survivors and Non-Survivors.

Outcome	Lymphocyte (Cells/MicroL)		
	Kruskal–Wallis H	df	*p*
Non-survivors	79.067	4	0.001
Survivors	5.477	4	0.242

**Table 11:** Mann–Whitney U Test Comparing NLR Between Survivors and Non-Survivors.

Outcome	Moment	N	Mean	Median	SD	Mann–Whitney U	Z	*p* (Two-Tailed)
D1	Non-Survivors	81	11.04	10.51	6.57	473.000	−0.987	0.324
	Survivors	14	14.45	11.49	9.93			
D3	Non-survivors	81	19.99	16.20	13.94	433.000	−1.407	0.159
	Survivors	14	15.02	13.25	10.92			
D5	Non-Survivors	79	27.92	25.56	15.52	396.500	−1.681	0.093
	Survivors	14	22.00	14.46	17.56			
D7	Non-survivors	72	31.02	29.76	16.75	236.500	−3.129	0.002
	Survivors	14	15.93	11.01	14.09			

**Table 12:** Within-Group Kruskal–Wallis Analysis of Temporal Changes in NLR in Survivors and Non-Survivors.

Outcome	NLR		
	Kruskal–Wallis H	df	*p*
Non-Survivors	92.123	3	0.001
Survivors	1.814	3	0.612

**Table 13:** Results of ROC analysis.

	AUC	SE	95% CI for AUC	z	*p*	J	X_k_	Se (%)	Sp (%)
CD4^+^ (cells/mm^3^)	0.734	0.2080	0.558 to 0.868	1.123	0.2616	0.5565	≤179.00	80.65	75.00
CD8^+^ (cells/mm^3^)	0.569	0.1580	0.391 to 0.734	0.435	0.6636	0.2903	≤51	29.03	100.00
CD4^+^/CD8^+^ (cells/mm^3^)	0.698	0.1450	0.519 to 0.841	1.367	0.1716	0.4032	≤2.44	90.32	50.00
Lymphocytes (cells/microL)	0.556	0.0828	0.450 to 0.658	0.676	0.4988	0.1834	>700.00	46.91	71.43
NLR	0.583	0.0942	0.477 to 0.683	0.880	0.3789	0.2460	≤18.37	88.89	35.71

AUC = area under the ROC curve; SE = standard error; z = test statistic; *p* = associated probability; J = Youden index (maximum vertical distance between the ROC curve and the diagonal reference line); Xk = cut-off value; Se (%) = sensitivity; Sp (%) = specificity.

## Data Availability

The original contributions presented in this study are included in the article. Further inquiries can be directed to the corresponding author.

## Abbreviations

The following abbreviations are used in this manuscript:

ALC	Absolute lymphocyte count
NLR	Neutrophil-to-lymphocyte ratio
ROS	Reactive Oxygen Species
TCR	T-cell receptor
SOFA	Sequential organ failure score
APACHE II	Acute Physiology and Chronic Health Evaluation II
BS	Bacterial Sepsis
